# Magnetic resonance imaging /ultrasonography fusion transperineal prostate biopsy for prostate cancer: Initial experience at a Middle Eastern tertiary medical centre

**DOI:** 10.1080/2090598X.2021.1926727

**Published:** 2021-07-14

**Authors:** Adnan El-Achkar, Mouhammad Al-Mousawy, Nassib Abou Heidar, Hisham Moukaddem, Hero Hussein, Nadim Mouallem, Albert El-Hajj, Muhammad Bulbul

**Affiliations:** a American University of Beirut Medical Center, Department of Surgery, Division of Urology, Beirut, Lebanon; b American University of Beirut Medical Center, Department of Diagnostic Radiology, Beirut, Lebanon

**Keywords:** Prostate biopsy, prostatic neoplasms, image-guided biopsy, fusion biopsy, transperineal biopsy

## Abstract

**Objective**: To report on the outcomes of magnetic resonance imaging (MRI)/ultrasonography (US)-fusion transperineal prostate (TP) biopsy at a tertiary medical centre in the Middle East including detection rate of clinically significant prostate cancer (csPCa), complications, and tolerability of the procedure.

**Patients and methods**: Between May 2019 and June 2020, 98 MRI/US-fusion TP biopsies were performed in the US suite using light sedation. All patients had pre-biopsy 3-T multiparametric MRI. Data on patient characteristics, PCa detection rate and complication rates were collected retrospectively. A Gleason score ≥3 + 4 was defined as csPCa.

**Results:**

There were 98 patients, with a mean (SD) age of 65 (9.1) years, and a median (SD) prostate-specific antigen level prior to biopsy of 7.53 (12.97) ng/mL and prostate volume of 51 (31.1) mL. PCa was detected in 54 (55%) patients, with csPCa detected in 43 (44%). A total of 124 Prostate Imaging-Reporting and Data System (PI-RADS) 3–5 lesions were targeted. Grade Group ≥2 PCa was found in 35.5% of the targeted lesions. Random biopsies detected one csPCa Gleason score 3 + 4 in one patient with a negative target. None of the patients had post-biopsy haematuria or retention. Only one patient developed acute prostatitis requiring in-patient intravenous antibiotics.

**Conclusions:**

MRI/US-fusion TP biopsy has an adequate detection rate of csPCa with minimal complications and low infection rates after biopsy. This is one of the first TP biopsy series in the Middle East paving the way for wider adoption in the region.

**Abbreviations:**

AS: active surveillance; AUR: acute urinary retention; GG: Grade Group; IQR: interquartile range; mpMRI: multiparametric MRI; (cs)PCa: (clinically significant) prostate cancer; PI-RADS: Prostate Imaging-Reporting and Data System; TP: transperineal; US: ultrasonography; TRUS: transrectal Ultrasound guided.

## Introduction

The standard TRUS 12-core systematic biopsy continues to be the most popular urological procedure to diagnose prostate cancer (PCa). It is estimated that a million prostate biopsies are taken yearly in the USA alone [1,2]. TRUS biopsies or ‘transfecal biopsy’ have shown a significant risk of UTI, which has been minimally mitigated with the use of augmented antibiotics. The risk of urosepsis remains substantial with rates as high as 3% reported [3]. One episode of sepsis in the USA could cost between 8672 USD and 19 USD 100 (American dollars) [2].

The transperineal prostate (TP) biopsy has been recently re-introduced into practice in many parts of the world and has effectively eliminated the risk of urinary infections and readmissions [4–6]. Dfgirect comparison between TRUS biopsies and TP biopsy showed a similar detection rate of clinically significant PCa (csPCa), with significantly lesser infection rates with TP vs TRUS biopsy [7–9]. The adoption of the TP approach has been cautious in the past; however, it has gained momentum in the last few years in the USA and elsewhere especially in the current era of fusion technology [10].

We have adopted TP biopsy in clinical practice as an office-based prostate-sampling modality considering patient safety and PCa detection rate. In the present study, we report the results of our experience with MRI/ultrasonography (US)-fusion targeted TP biopsy at a single tertiary care centre.

## Patients and methods

### Patients and data analysis

Between May 2019 and June 2020 retrospective data were collected of 98 patients that underwent TP biopsy at our institution. Clinical data regarding age, prostate volume, PSA level, medical and surgical history, as well as MRI results prior to biopsy were collected. Outcomes including pathology report and complications, such as acute haematuria, sepsis and acute urinary retention (AUR) were collected as well. Medians and interquartile ranges (IQRs) were reported for continuous variables and counts and percentages were used for categorical variables. The Statistical Package for the Social Sciences (SPSS®) for MAC OS, version 25 (IBM Corp., Armonk, NY, USA) was used to report the results.

All patients had multiparametric MRI (mpMRI) prior to biopsy. A PIRADS score was used for classification of lesions on MRI and a Gleason score of ≥3 + 4 was used as a definition for csPCa. Patients’ comfort after the procedure was measured using the Wong–Baker faces pain scale [11].

### Biopsy preparation

The procedures were performed in the clinic under light intravenous sedation at American University of Beirut Medical Center. The team comprised a urologist, radiologist, one nurse and the anaesthesia team. After signing the consent, the patient was put into light sedation while in a supine position. After proper sedation, the patient was transferred into a lithotomy position. The scrotum was elevated away from the perineum using micropore tape. The perineum was then prepped and draped using betadine and sterile drapes. The Koelis Trinity®: MRI/US organ-based tracking (OBT)-fusion system was used (Koelis, Meylan, France). The bi-planar US probe was advanced into the rectum after being clamped on the stepper known as the steady PRO probe holder. The biopsy perine full grid was set up on the stepper against the perineum. The grid guides the operator ensuring accurate positioning and targeting of lesion. Antibiotics such as ciprofloxacin 500 mg (one tablet) was taken by the patient the morning of the procedure. No fleet enema was requested to be taken by the patient prior to procedure. Local anaesthesia was applied using 20 mL xylocaine 2% on the perineum.

### MRI/US fusion-guided and targeted biopsy

After introduction of the transrectal US probe, the prostate edges were contoured and the images of the mpMRI were superimposed on the US images. This was done by two of our expert uro-radiologists. The suspicious lesions were then easily identified and defined on the three-dimensional prostate shape. Targeted and random needle biopsies were taken. Live tracking of biopsy locations with live US were displayed on the screen and saved. The targeted cores were taken, as well as random cores targeting uninvolved zones of the prostate.

## Results

Between May 2019 and June 2020, 98 patients underwent TP biopsy at our centre using the described method. Patients had a median (IQR) age of 64.5 (59–72) years and a serum PSA level of 7.53 (4.79–13.7) ng/mL. A median (IQR) number of 11 (9–14) cores were taken including targeted and random cores; a median (IQR) of 11 (9–12) targeted and 3 (2–6) random cores were taken. A median (IQR) of 6 (4–7) and 5 (4–6) cores were taken from the index and secondary lesions, respectively. A total of six patients (6.12%) were on active surveillance (AS), while 92 (93.8%) were biopsied due to a rise in PSA level and presence of suspicious lesion(s) on mpMRI. There was an abnormal DRE in 42 patients (42.9%).

A total of 124 Prostate Imaging-Reporting and Data System (PI-RADS) 3–5 lesions were targeted during biopsy and 37 patients (37.7%) had two or more lesions on MRI. The number of PI-RADS 3, 4 and 5 lesions were 27 (21.7%), 71 (57.2%) and 26 (20.9%), respectively. Detailed characteristics of the cohort are listed in Tables 1 and 2.

**Table 1.** Demographics of the patient population undergoing TP biopsy

Variable	Value
Number of patients	98
Age, years, median (IQR)	64.5 (50–72)
PSA level, ng/mL, median (IQR)	7.53 (4.79–13.7)
Prostate volume, mL, median (IQR)	51 (40–68.7)
Indication for biopsy	93% rise in PSA, 6% AS
History of TURP, *n*	17
On 5α-reductase inhibitors, *n*	10
Size of the dominant lesion, cm, median (range)	1 (0.7–1.5)
Clinical stage, *n* (%)	
cT1c	52 (53.1)
cT2a	43 (44.3)
cT2b	2 (2)
≥cT2c	0 (0)
cTx	1 (1)
PI-RADS scores of MRI targets, *n* (%)	
3	27 (21.7)
4	71 (57.2)
5	26 (20.9)
Number of MRI targets per patient, median (IQR)	1 (1–2)
Highest overall GG, *n* (%)	
No PCa	39 (39.8)
GG1	11 (11.2)
GG2	15 (15.3)
GG3	13 (13.3)
GG4	5 (5.1)
GG5	12 (12.2)

**Table 2.** Demographics of patients with GG ≥2 PCa vs non-significant biopsy including those that had no PCa and non-significant PCa (GG <2 PCa)

Variable	GG ≥2 PCa (*N* = 43)	Non-significant biopsy (*N* = 54)
Age, years, median (IQR)	72 (65–74)	61.5 (55–66)
PSA level, ng/mL, median (IQR)	9.0 (5.6–16.4)	7 (4.6–11.2)
Prostate volume, mL, median (IQR)	46.5 (38–73)	54 (42–65)
Abnormal DRE, %	51.2	37
History of TURP, *n* (%)	9 (21)	8 (14.8)
On 5α-reductase inhibitors, *n* (%)	5 (12)	5 (9.3)
Size of the dominant lesion, cm, median	1.25	0.9
Number of cores fired on target, median	5	6
Total number of cores fired including random, median (IQR)	9 (8–13)	12 (10–14)

**Figure 1.**
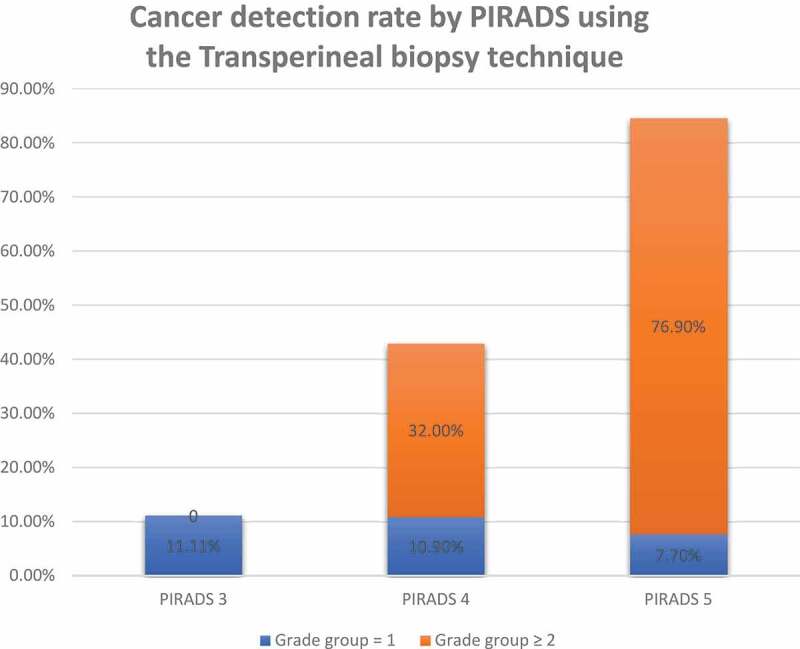
PCa detection rate by PI-RADS 3–5 using TP biopsy

In total, 54 patients (55%) were diagnosed with PCa, with csPCa detected in 44%. Of the 124 targeted lesions, the detection rate for Grade Group (GG) >1 PCa was 47.6%, while for GG ≥2 it was 35.5%. As the PI-RADS lesion score raised the detection rate of csPCa increased (Figure 1). For patients with PI-RADS 3 lesions, none had csPCa. For those with PI-RADS 4 and 5 lesions, the detection rate of csPCA was 32% (23/71) and 76.9% (20/26), respectively.

In all, 88 patients (90%) had random biopsies taken in addition to targeted biopsies. Out of those, 75 (85.2%) patients had negative random biopsies and eight patients (9%) had csPCa detected in random cores. Only one patient had GG ≥2 PCa on random biopsy with a negative target. The detection rate of PCa when targeting dominant lesions at the apex was 62% (10/16) and was 54% (12/22) for dominant lesions located anteriorly. A total of 21 patients underwent robot-assisted radical prostatectomy after biopsy. One patient upstaged (≥pT3), seven patients were upgraded, and five were downgraded.

Notably, none of the patients reported any discomfort or significant pain after the procedure. Of the 20% who had had a previous TRUS biopsy, all reported a preference for the TP approach. Using the Wong–Baker faces pain scale, patient reported a median (range) baseline pain level of 0 (0–2) after the procedure. None of the patient reported any retention, haematuria or haematochezia after biopsy. Only one patient (1.02%) developed cultured-confirmed acute prostatitis requiring in-patient intravenous antibiotics.

## Discussion

To the best of our knowledge, this is the first Middle Eastern report of MRI/US-fusion TP biopsy. The present study elucidates the adoption of TP fusion prostate biopsies at our institution. The key aspects of our techniques are the use of light sedation, the presence of a grid on a stable stepper, and a mounted real-time bi-planar US probe, which all contribute to a stable prostate and accurate targeting of lesions during biopsy. The added benefit of prostate MRI prior to biopsy is well studied in the literature [12–14]. The PROstate MRI Imaging Study (PROMIS) and PRostate Evaluation for Clinically Important Disease: Sampling Using Image-guidance Or Not? (PRECISION) trials underscored the importance of MRI prior to biopsy, showing it decreases the number of unnecessary biopsies and increases the detection of csPCa [12,15,16].

**Figure 2.**
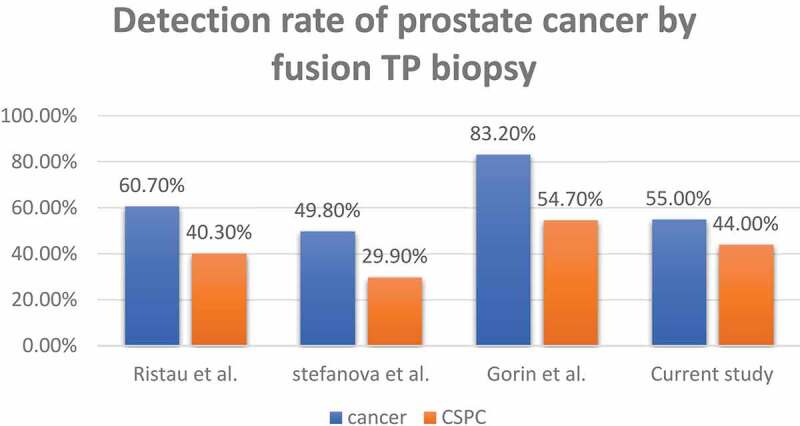
Detection rate of PCa and csPCa using fusion-TP biopsy in the present study in comparison to international series (Stefanova et al. 2019 [5], Ristau et al. 2018 [15] and Gorin et al. 2020 [17])

Our present PCa detection rate is comparable to that reported in other studies (Figure 2) [5,15,17]. Although, Gorin et al. [17] reported a higher detection rate (54.7% vs 44%) of csPCa, this is probably due to our present cohort having significantly fewer patients on AS (6.12% vs 41.1%). When comparing csPCa detection rate by PI-RADS, we had a similar detection rate for PI-RADS 5 lesions (76.9% vs 76–79.5%) and a slightly lower detection rate of csPCA for PI-RADS 4 lesions (32% vs 38–44%) [17–20]. None of our targeted PI-RADS 3 lesions had csPCa, while most studies report a detection rate of 8–19% [17,18,19,20,21,22]. Several strategies of combining additional information regarding lesion size, PSA density and biomarkers may be beneficial in stratifying PI-RADS 3 lesions into high- and low-risk lesions in order to maximise detection rate of csPCa and minimise unnecessary biopsies [23,24]. In our present cohort, the lack of csPCa is partly due to selection bias due to a small sample size, as well as possible over reading of low-risk MRI lesions as PI-RADS 3. Lastly, the TP biopsy was excellent for targeting lesions in the anterior zone (54%) and apex (64%), recent literature has shown that TP approach is superior to TRUS in accurately targeting these difficult areas [25].

In biopsy-naïve patients the detection rate of PCa with randomised non-targeted biopsy is 20–30% [26,27]. Some studies have shown a superior PCa detection rate when TP-targeted biopsy is combined with systematic biopsy [28]. However, the detections rates of csPCa did not rise significantly with the addition of non-targeted biopsy cores [29]. The debate regarding the benefit of adjunct non-targeted core to targeted core remains open. Only one patient had a Gleason Grade 3 + 4 lesion on random core biopsy with a negative target core in our cohort. These results reinforce the findings of the multicentre study by Miah et al. [18] that showed that there is limited clinical values in adding non-targeted (or random) biopsy cores to unsuspicious areas due to the low yield of csPCa.

As previously mentioned, the TP route avoids inoculation of rectal bacteria into the prostate that would otherwise be inevitable by the classic transrectal route. According to the European Randomized Study for Screening for Prostate Cancer trial, TRUS biopsy conferred a 4% risk of febrile UTI and a 1% risk of hospital admission [30]. A recent meta-analysis has shown that the infectious complications of TRUS biopsies are on the rise with readmissions reaching 6% in some series [31]. On the other hand, TP biopsies have nearly eliminated this risk [4]. In our present cohort, only one patient had a febrile UTI requiring hospitalisation for parenteral antibiotics. Cost–benefit stratification needs to be performed to examine how impactful these febrile UTIs are after a prostate biopsy.

One hindrance to the widespread adoption of TP biopsy, is the issue of outpatient feasibility whereby many centres have performed the biopsy under general anaesthesia while occupying an operating room [32,33]. However, many urologists are currently performing these biopsies under local anaesthesia [17]. We perform the TP biopsies in a dedicated US suite with the help of a dedicated radiologist as an outpatient procedure. Our TP biopsies are done with light sedation and we have found it to be a successful form of analgesia for the patients, as well as an excellent method to further decrease the motion of the patient’s prostate for better fusion targeting. One advantage of light sedation over pure local anaesthesia is the absence of pain experienced from the first pricks of local anaesthesia or during the procedure [17]. In fact, all of the patients that had a previous TRUS biopsy reported less pain in the TP biopsy and the reported pain scores of our present cohort are even lower that those that had had TP biopsy with only local anaesthesia [17]. Moreover, sedation offers the advantage of possibly decreasing the incidence of post-procedure AUR due to the omission of the periprostatic block [34]. Post-biopsy AUR has been mentioned as one caveat of the TP approach, whereby retention was unusually high at 24% in the PICTURE trial (ClinicalTrials.gov Identifier: NCT01492270) [7], where the biopsy was done under general anaesthesia, while others reported a urinary retention rate of about 0–2% when done under local anaesthesia [5,35]. As Pepe and Aragona [36] and Buskirk et al. [37] have shown, a higher number of cores predisposes to more complications including more haematuria and a higher AUR rate. We can postulate that general anaesthesia, periprostatic block and an extensive of number cores taken (>12 cores) could explain the higher AUR rate in some studies.

One of the hurdles for the adoption of fusion-TP biopsy is the cost of light sedation and the fees for the anaesthesiologist, the authors believe that this added cost is mitigated by the near-elimination of post-biopsy UTIs [9,38,39] and the decrease use of prophylactic antibiotics, pre- and post-biopsy, as well as the decrease rates of AUR.

Another issue that would limit the implementation of the TP route is the perceived added time. We did not experience this matter after overcoming a very small learning curve. In fact, recent reports point towards adequate prostate sampling using the TP route in ~10 min [35].

The limitations of the present study include the retrospective nature of the study and the small sample size. Another limitation is the lack of a control cohort in the study design. Future randomised controlled comparative studies comparing TRUS vs TP are needed to establish its superior detection rate and clinical effectiveness. In our present study, no objective questionnaire was administered to the patients to assess their self-reported pain, haematuria, or haematochezia. This could be considered a reporting bias and is one of the limitations of the study. Nevertheless, to our knowledge this is the first series reporting on outcomes of fusion-TP biopsy in the Middle East, with promising results.

With a comparable detection rate of csPCa, low infection rate, office-based practicality and less use of prophylactic antibiotics pre- and post-biopsy all have shifted the advantage towards the TP approach. Our present study confirms the safety and efficacy of TP biopsy as an office-based procedure. The MRI/US-fusion TP biopsy allowed an overall PCa detection rate of 55% and csPCa detection rate of 44% with minimal complications.
